# Family and community concerns about post-mortem needle biopsies in a Muslim society

**DOI:** 10.1186/1472-6939-12-10

**Published:** 2011-06-13

**Authors:** Emily S Gurley, Shahana Parveen, M Saiful Islam, M Jahangir Hossain, Nazmun Nahar, Nusrat Homaira, Rebeca Sultana, James J Sejvar, Mahmudur Rahman, Stephen P Luby

**Affiliations:** 1 GPO 128, International Centre for Diarrheal Diseases Research, Bangladesh (ICDDR,B), Mohakhali, Dhaka 1000, Bangladesh; 2 Centers for Disease Control and Prevention, Division of Viral and Rickettsial Diseases, National Center for Zoonotic, Vectorborne, and Enteric Diseases, 1600 Clifton Rd, MS A-39, Atlanta, GA, 30333, USA; 3 Institute for Epidemiology Disease Control and Research (IEDCR), Ministry of Health and Family Welfare, Government of Bangladesh, Mohakhali, Dhaka 1000, Bangladesh

**Keywords:** post-mortem, Bangladesh, needle biopsy, outbreak, diagnosis, informed consent

## Abstract

**Background:**

Post-mortem needle biopsies have been used in resource-poor settings to determine cause of death and there is interest in using them in Bangladesh. However, we did not know how families and communities would perceive this procedure or how they would decide whether or not to consent to a post-mortem needle biopsy. The goal of this study was to better understand family and community concerns and decision-making about post-mortem needle biopsies in this low-income, predominantly Muslim country in order to design an informed consent process.

**Methods:**

We conducted 16 group discussions with family members of persons who died during an outbreak of Nipah virus illness during 2004-2008 and 11 key informant interviews with their community and religious leaders. Qualitative researchers first described the post-mortem needle biopsy procedure and asked participants whether they would have agreed to this procedure during the outbreak. Researchers probed participants about the circumstances under which the procedure would be acceptable, if any, their concerns about the procedure, and how they would decide whether or not to consent to the procedure.

**Results:**

Overall, most participants agreed that post-mortem needle biopsies would be acceptable in some situations, particularly if they benefitted society. This procedure was deemed more acceptable than full autopsy because it would not require major delays in burial or remove organs, and did not require cutting or stitching of the body. It could be performed before the ritual bathing of the body in either the community or hospital setting. However, before consent would be granted for such a procedure, the research team must gain the trust of the family and community which could be difficult. Although consent may only be provided by the guardians of the body, decisions about consent for the procedure would involve extended family and community and religious leaders.

**Conclusions:**

The possible acceptability of this procedure during outbreaks represents an important opportunity to better characterize cause of death in Bangladesh which could lead to improved public health interventions to prevent these deaths. Obstacles for research teams will include engaging all major stakeholders in decision-making and quickly building a trusting relationship with the family and community, which will be difficult given the short window of time prior to the ritual bathing of the body.

## Background

A primary goal of public health is to prevent premature deaths, which requires specific knowledge about the proximate causes of death in a population. In Bangladesh, most deaths cannot to be attributed to any specific etiology because many patients die at home without accessing the formal healthcare system [1-5]. Even when care is sought, infrastructure for collecting and testing diagnostic specimens is inadequate. Physicians may also be reluctant to collect specimens from severely ill patients for fear that the procedure may be seen by families as contributing to the death [6]. An outbreak of sudden child deaths during 2008 went undiagnosed, in part, because children were either dead when they arrived at the hospital or died before specimens were collected. (ES Gurley, unpublished data)

Current approaches to investigate why people die in Bangladesh include hospital or community-based surveillance studies [7-13] to determine etiology of severe disease, or population-based verbal autopsy studies [14-18]. These approaches, however, have important limitations. Hospital-based studies capture only patients who access care and who, as such, may be more likely to survive illness; both hospital and community-based surveillance strategies are more likely to identify patients surviving illness from a particular etiology rather than deaths [9,19,20]. In addition, in some cases, the presence of a particular infectious organisms in clinical specimens may not necessarily represent the pathogens responsible for death [21-24]. Verbal autopsy studies capture deaths occurring at facilities and in the community, but the non-specific nature of diagnoses assigned by verbal autopsies [25,26] can be a barrier to development of specific public health interventions, particularly for deaths from infectious causes. Many of the most effective interventions, such as vaccines, are pathogen specific and therefore require etiologic diagnosis of death.

A direct approach to characterizing cause of death, which could be used during surveillance or outbreak investigations, are post-mortem exams; this approach focuses on patients who actually die and could provide specimens obtained by invasive methods for diagnosing etiology of deaths, particularly those caused by infections. The diagnostic value of post-mortem specimens for this purpose is well described [27-33]. Autopsies are routinely conducted in Bangladesh as a part of police investigations into possible homicides, suicides, or other unnatural deaths but not outside of this context [34].

Although Islamic texts do not directly address the issue of autopsy, a well known quotation by the Prophet Muhammad states, "Breaking the bone of a dead person is akin to breaking the bone of a living person", and some interpret this to mean that dead bodies feel pain as acutely as the living, which makes autopsy a cruel, and therefore unacceptable, procedure [35]. Rispler-Chaim outlines additional concerns about autopsy from the Muslim perspective: autopsy delays burial and the body must be buried as soon as possible after death; organ removal violates the sanctity of the human body; and autopsy requires that the body be moved to and from the autopsy site, which is undesirable because it can further desecrate the body [35]. Nonetheless, some Egyptian and Syrian Muslim scholars have issued legal opinions (called *fatwas*) proposing that if there is a compelling reason to perform an autopsy, including benefit to society or prevention of disease, then these religious commands may be suspended [35].

Given the potential value of post-mortem specimens in diagnosing cause of illness and death and possible objections to autopsy in predominantly Muslim Bangladesh, we propose the collection of post-mortem needle biopsies to aid in diagnosing cause of death. Post-mortem needle biopsies have been collected in some settings for decades [36-39], including in settings where families may object to full autopsy, such as Kuwait [40]. Although inferior to full autopsy [41], post-mortem biopsies have been shown to be valuable in diagnosing cerebral malaria and Japanese encephalitis, two significant contributors to morbidity and mortality in low-income Asian countries like Bangladesh [42-44]. Needle biopsies might be preferable to autopsy because the body would not be cut, no organs removed, and because the procedure could be conducted more quickly, burial would not be delayed. However, we were unsure how families and communities would perceive this procedure or how they would make decisions about whether or not to participate in research that included post-mortem biopsies. The goal of this study was to better understand family and community concerns and decision-making about post-mortem needle biopsies in this low-income, predominantly Muslim country in order to determine the feasibility of public health research studies using this approach and design an appropriate informed consent process.

## Methods

We selected five communities that experienced between 2 and 14 deaths during a Nipah outbreak in Bangladesh during 2004-2008 for participation in this study [45-47]. Selected communities were located within approximately 100 km of each other (about a 5 hour trip by car). These communities were chosen because they experienced outbreaks associated with high case fatality rates and we were interested in using post-mortem needle biopsies in similar outbreak settings.

During the Nipah outbreaks members of our outbreak investigation team visited affected communities to find cases, collect specimens from sick persons, conduct risk factor studies, describe illness and exposure histories, and provide the community with information about Nipah virus, its transmission, and how to prevent its spread. The investigation team was usually present in outbreak communities for weeks, and follow-up visits were often made to evaluate communication campaigns [6] or continue medical follow-up with survivors [48]. Intimate and prolonged interactions between researchers and villagers resulted in close relationships between researchers and communities. We anticipated more open and honest responses to our questions within the context of this established relationship with study communities.

Families were approached in their homes by the research team about participation in the study. Qualitative researchers with experience working with families during fatal outbreaks approached the immediate family members of the deceased and told them that they were conducting a research study about finding out the cause of death during outbreaks. Researchers asked family members when would be a good time to visit them again to discuss the study and their potential participation. The study team returned at a date and time convenient for the families.

First, qualitative researchers conducted group discussions with eight families who had one or more family members who died during these outbreaks. (Table 1) We chose families for participation based on their continued residence in the outbreak community and the age and sex of the patient who died during the outbreak; we wanted to have input from families who lost both male and female adults and children to investigate any differences in concerns or opinions by age and sex of the deceased. Only families affected by outbreaks more than one year prior to the study were eligible to participate to minimize possible psychological risks to participants in discussing the death of loved ones. Bangladeshi researchers trained in qualitative methods with expertise in eliciting responses about sensitive topics led the group discussions. One group discussion was held separately for male and female family members of each decedent with researchers of the same sex leading the discussions. Discussions were sex segregated because of we believed that unequal power relationships between men and women in Bangladesh could hinder women's participation in a discussion [49]. (Table 1)

**Table 1:** Group discussion participants and key informant interviews completed by village and family with demographics of deceased, Bangladesh, 2009

				
	**Age and sex of ** **deceased**	**No. participants in ** **female group ** **discussion**	**No. participants in ** **male group discussion**	**No. and type of key ** **informant interviews**

Village 1				
Family 1	5 years, male	9	5	
Family 2	22 years, male	4	5	
				1 community leader
				1 religious leader
				

Village 2				
Family 3	12 years, male	6	8	
				1 community leader
				1 religious leader
				

Village 3				
Family 4	60 years, male	6	6	
	35 years, male			
	8 years, male			
Family 5	45 years, male	3	3	
Family 6	40 years, female	5	8	
	32 years, male			1 community leader
	15 years, male			2 religious leaders
				

Village 4				
Family 7	35 years, female	5	5	
				1 community leader
				1 religious leader
				

Village 5				
Family 8	7 years, male	8	6	
	15 years, male			
				1 community leader
				1 religious leader

Researchers began the group discussions by describing to respondents the difficulties with diagnosing cause of death in Bangladesh and the potential public health benefits of finding new tools to diagnose cause of death. Then, the post-mortem needle biopsy procedure was described for families and researchers asked them whether they would have agreed to this procedure when their loved one(s) died. Families were shown a vial containing a small amount of chicken meat to help the respondents understand the amount of tissue which would be removed during the needle biopsy. Researchers told participants that they would be interested in collecting tissue from the brain, liver, and lung; a drawing of the procedure to collect a brain specimen through the eye was shown to families on request. Researchers asked probing questions about the circumstances under which the procedure would be acceptable, if any, their concerns about the procedure, and how the decision to participate in post-mortem biopsy would be made by the family. Specifically, we asked respondents about their own feelings of acceptability and also asked them to speculate about possible objections that other community members may have. We asked for families' advice about how to approach other families in future outbreak settings. Respondents were asked to name any community or religious leaders they consulted during the outbreak or might have consulted during their decision making process had they been asked to provide consent for the procedure.

Researchers made a list of the names of community and religious leaders from whom families sought advice during the outbreaks and approached these leaders to participate in key informant interviews. These community and religious leaders were important to include in the study because they often advised families on healthcare seeking and were involved in responding to outbreaks as community crises. Key informant interviews began with researchers presenting the study rationale and post-mortem needle biopsy procedure, similar to the way it was presented to families. Then we asked leaders to recall their experiences during the Nipah outbreak in their community and to describe how they would have advised families about consenting to post-mortem needle biopsies had they been asked to provide input during the outbreak. Five community and six religious leaders were interviewed for this study. (Table 1)

All group discussions and key informant interviews were tape recorded and researchers took detailed notes during data collection about the number of participants in each group, their relationships with the deceased, and overall impressions about group participation. The audio recordings were transcribed into Bengali and then translated into English using field notes to provide context. Portions of the interviews where little information was provided were summarized rather than transcribed in full. Direct quotations illustrating key study themes were translated verbatim from audio files. We used a thematic analysis [50] to summarize respondents' major thoughts and concerns about post-mortem needle biopsies, including acceptable motivations for conducting the procedure, issues of trust, religious and social concerns, emotional concerns, and the consent and the decision making process. Themes for the analysis combined those decided during the design of the study and those that arose during interviews with participants. Next, we manually coded and summarized responses according to these major themes.

### Human subjects protection

Study participants provided informed, written consent prior to participation in this study. Participants who were unable to write their names were asked to provide a thumbprint to symbolize their consent to participate. ICDDR,B's ethical review committee reviewed and approved this protocol.

## Results

All participants approached for this study agreed to participate. All but a few study participants said that they would agree to post-mortem needle biopsies for diagnosing cause of death, although they named numerous concerns that would need to be addressed by researchers prior to participation. (Table 2) Responses concerning religious, social, and logistical concerns about the procedure were remarkably consistent between outbreak communities. The most important concerns for families and community and religious leaders alike included the motivation for conducting the procedure, such as perceived benefits for their community and the broader society, trust in the research team and their intentions, and religious or social prohibitions. (Table 2)

**Table 2:** Family and community concerns about post-mortem needle biopsies, Bangladesh, 2009

Will this procedure have a benefit to society?
Do we value a medically diagnosed of cause of death?
Do we trust the physician and research team?
Do we believe that the physician and researchers have our best interests in mind?
Does Islam prohibit this kind of procedure?
Does the government support this procedure?
Will the procedure remove any organs from the body?
Will the body feel pain from the procedure?
Will the procedure result in substantial delays in burial?
Will female physicians collect specimens from adult female bodies and male physicians from adult male bodies?
May we elect to have someone we trust to observe the procedure?

### Acceptable motivations for conducting the procedure

Respondents agreed that post mortem needle biopsies would only be acceptable if there were a compelling reason to perform them. For most respondents, the possibility of learning more about a disease, and thus being better able to prevent it, would be a major motivation for consenting to a post-mortem needle biopsy. This perceived societal benefit was highly valued, particularly in the case of a lethal outbreak involving numerous deaths, and particularly if respondents believed that the procedure could prevent future deaths in their community. As one male respondent said,

*"It's quite possible (to collect these biopsies). It is rather good for us. For example, many people died from cholera earlier, but nobody dies now. The doctors have driven away this disease. According to my view, this work is done for a noble cause that is why it is possible. As it can save many lives, it is good for us."*(Male group discussion, family 6)

Some family members believed that learning the cause of death of a loved one would be intrinsically valuable, but others disagreed, particularly because of the belief that the timing of death is determined by God. Respondents believed that families might be more reluctant to agree to the procedure when the cause of death was known, such as from a car accident, or if the death was "expected", as in the case of the elderly. As one community leader said,

"*In case of the death of an elderly person it would not be possible for you to collect the specimen. This is because the family members would assume that the person had died naturally by the burden of age; so what would be the benefit of knowing the cause of his/her death?"*

### Building trust

Respondents reported that trust in the research team would be a major consideration in their decision to agree to the procedure. Researchers were seen as cultural outsiders and therefore their motivations for collecting the specimens were suspect. Families were concerned that the research team may remove body parts which would be sold for profit or that the research team was motivated to collect the specimen for some unknown personal gain. As one man said,

*"People in other areas might not agree to this (post-mortem biopsy). They might object. They might suspect that you would take some organs from the body. We know you but people from other areas don't know you so they might think in this way." *(Male group discussion, family 4)

They suggested that research teams be accompanied by local physicians or community leaders when they approach families in the community and that they bring documentation of support from the Government of Bangladesh for conducting the procedure.

*"You (research team) can come to the village with a thana *(sub-district) *health complex doctor who is familiar to residents or renown in this area, or with a village doctor. Then the doctor will talk with the people of the area. If the people of the area don't want to trust you after this discussion with them then you should show them your official document and identification card. Then they will be confident in where you came from." *(Female group discussion, family 8)

Transparency about the proposed procedure was paramount and families suggested that allowing a family representative to observe the procedure could promote trust.

"*I will believe only that what will takes place in front of my two eyes... everything else is unbelievable"*. (Female group discussion, family 7)

Respondents reported that sometimes trust can be broken during interactions with the medical system during the patient's illness. Some believed that if the medical system was unable to save the patient's life then they would not trust this same system with handling the body after death. Some family members recalled that during the outbreak, medical procedures such as lumbar puncture were conducted without their consent or without explaining the purpose of the procedure to the family and that this damaged their trust of the medical system. These sentiments were also reported during a previous study following a Nipah virus outbreak during 2004 [6].

### Religious and social considerations

Neither religious leaders, community leaders, nor family members reported that post-mortem examination was prohibited by Islam. Many religious leaders told us that if the procedure would help others then it should be encouraged by the Muslim community; one leader offered to promote acceptance of the procedure during his weekly sermon to the community. Another religious leader said,

"I will suggest them to give the specimen as it has no harm; it will help you and others of the country. If we can learn the cause of death through this research, then it will be good for all."

However, all respondents mentioned religious and social considerations which would be important in decision making and determination of whether or not the procedure was acceptable. All respondents voiced their concern that bodies be treated with the utmost respect and care. Some believed that this was a religious imperative and others believed that it was purely a social consideration. Many respondents believed that the deceased could still feel physical pain and they were concerned that the procedure might cause further pain to someone who had already suffered during illness. The extent to which the body would be touched or intervened upon was a key concern for participants. They reported that full post-mortem autopsy was unacceptable because organs were removed from the body and the procedure left cuts and stitches on the body. Participants believed that a post-mortem needle biopsy would be more acceptable because there would be no visible cuts to the body and because no organs would be removed. The small amount of tissue which would be removed during the procedure was a major part of the procedure's acceptability. As one community leader explained:

"If I donate my eyes after my death then a big part of my body will be lost. This procedure will take a small piece (of tissue) for testing so everybody should give this."

**Figure 1:**
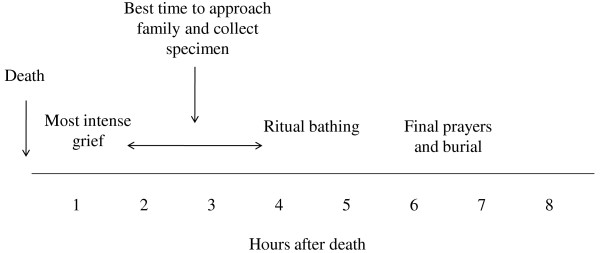
**Window of opportunity for collection of post-mortem needle biopsies in the typical Muslim, Bangladeshi funeral and burial process**.

There were numerous religious and social customs surrounding burial that must be respected as part of the biopsy process. Muslim families desire to bury the body as soon as possible after death and so the procedure should ideally minimize any delay in burial. In addition, respondents unanimously reported that the procedure would only be acceptable prior to the ritual bathing of the body, which typically occurs within a few hours of death, and that the sex of the physician collecting the biopsy should be the same as the body in the case of adult death. The window of opportunity for collecting biopsies was short, but well-described by respondents. (Figure 1) One female family member summarized the recommendations of many families:

*"When a person dies, the body will be buried within five or six hours after death. If you (the research team) come within one hour (of death), you can't collect the specimens because then everyone in the house will be crying and mourning. If you come after one or two hours then the relatives will be more approachable. If you will come then and discuss the procedure with them for half an hour or one hour then they will give you permission." *(Female group discussion, family 8)

Although no respondents believed that the procedure was prohibited by Islam, many wanted to know what religious leaders thought of the procedure and said that they would consult a religious leader as part of their decision-making. One religious leader also reported that he was unsure about Islamic teachings on post-mortem exam and suggested that the research team bring some documentation that the procedure is not prohibited by Islam. He believed that this kind of resource would make families and religious leaders more comfortable with making decisions about whether or not to allow specimens to be collected.

### Mothers of deceased children

Mothers who had lost children during the outbreak sometimes reported that their emotions would prevent them from agreeing to the procedure. These were the greatest objections to the procedure reported during any of the discussions or interviews. Some respondents reasoned that because the mother bears the child and performs most of the work of raising the child that their emotional pain after death was the greatest. Some mothers explained that because of their intense grief they would or should be removed from the decision making process to protect them from additional psychological trauma. As one mother explained:

*"I am a mother, so, I know the pain of losing my child. So, no one who is like me will allow specimens to be taken from her child's dead body. A mother like me would not be allowed to stay near the dead body and should be persuaded to keep away from the body. The specimen should be collected without our knowledge." *(Female group discussion, family 4)

Another mother reported that she would not have given permission for the procedure because it could not help her deceased child. She said,

*"*W*hen doctors took water from the bone (lumber puncture) of my son, then I didn't resist because they might save him but after death there is no hope of being well so I will not give permission to do it"*. (Female group discussion, family 6)

### Consent and decision making

Respondents all agreed that the body's guardian(s) must provide consent to the procedure before the specimen can be collected. In the case of adults, the guardian would likely be the male head of the family or the next of kin male family member. In the case of children, most agreed that the guardians would be the parents. However, if the parents disagreed about whether or not to provide consent, the father's wishes would take precedent. As one woman explained:

*"He who is the main head of the family will give the final decision. He can be the head of the family or can be the community leader in some special circumstances. If a child died and its mother does not agree to give permission to do this procedure but its father agreed then the father's decision will be honored as he is the begetter." *(Male group discussion, family 3)

Respondents described that families are a part of a society and therefore the family must have been reassured that the procedure will be acceptable in their society before they agree to it. They believed that the role of the researchers should be to engage various representatives from this society, including other family members and community and religious leaders, in a discussion so that the guardians can feel confident in their decision-making.

Responsibility for raising the child, deciding to seek medical care, payment for medical bills, and arranging for burial are responsibilities often shared by multiple family members. Deciding whether or not to provide the needle biopsy specimen would also be a shared decision making process. As one male respondent told us:

*"I was the patient's guardian and signed for his hospital admission though I am his uncle (his father's sister's husband). If you told me (about the procedure) then I would discuss with his (the patient's) father for permission." *(Male group discussion, family 2)

Guardians must be the ones to provide consent for the procedure. However, they should be approached gently because they are also likely to be most psychologically affected by the death. Ideally, respondents explained, more distant relatives or community leaders should be the ones to approach the grieving families about consenting for the procedure. These people already have a direct relationship with the family and were seen as the best ones to propose the procedure to the guardians. Therefore, the research team was encouraged to approach other family and community members about collecting the specimens prior to approaching the guardians.

Likewise, both leaders and families reported that guardians would usually seek out the advice of religious and community leaders during decision making. They suggested that this was further reason for the research team to approach these leaders from the beginning. Responses suggest that families with lower social standing may seek out advice from others more frequently. One community leader told us:

*"The poorest families in a community will accept the decision made by their *mattobbor *(community leader). On the other hand, rich families will usually make this decision by themselves. Some rich families might consult the *mattobbor *about this procedure. They may even say that if the *mattobbor *gives permission, they will not object (to the procedure). So, you will discuss this procedure with the *mattobbor, *as well as with the guardian of the dead person."*

Religious and community leaders are sought out as supplemental decision makers because their opinions are believed to be valuable in deciding whether the benefits of the procedure are compelling, if the team should be trusted, and if the procedure would be acceptable in the eyes of God. The head of one family told us:

"*For my family, I will do what the *hujur *(religious leader) will accept; but in some families, the *hujur *will do what the families will accept. In the views of Islam, we will accept what the *hujur *will decide, because we believe that the *hujur *knows (about religious rules) more than common people." *(Male group discussion, family 2)

Another community leader suggested that during outbreaks, the potential benefit to the community of learning the cause of death meant that community leaders were stakeholders in the decision making process, not just counselors, and therefore should be involved in swaying the opinions of the family. He said:

"The family could object. They could say that my loved one has died and we will not let you take a specimen. But the community may want to know why she or he died. So the community should be active (in decision making) in this kind of situation. That means that the community leader, who has the community's best interests at heart, should be involved in convincing the family to provide consent."

The stakeholders in decision making would likely also vary by location. Where fewer extended family members and community leaders were available, in the case of hospitalization far from home or when families migrate to the city, guardians may be entirely responsible for decision making.

### Conducting the procedure in the hospital or in the community

Respondents reported that the procedure could be performed either in the community or the hospital and the decision on where to conduct the procedure should be made on the basis of where the body was at the time the research team arrived. Most respondents agreed that the procedure would be more acceptable in the hospital because a family who had sought care in a hospital might be more open to medical intervention. However, some respondents also mentioned that families may feel powerless to refuse the procedure in the hospital. One community leader said:

"The guardian (of the body) would not be able to object if the doctors at the hospital say that they want to examine the dead body."

Once the body has been transported back to the community, families would not agree to move the body back to the hospital to conduct the biopsy because this could delay burial. In this scenario, or when death occurs in the community, then the procedure should be carried out there to minimize the impact of the procedure on the burial process. Respondents suggested partitioning off a room in a house with a curtain to create a semi-private location for the biopsy procedure.

## Discussion

In places like Bangladesh where the cause of death is frequently not identified, family and community acceptance of this procedure, at least during outbreaks, represents an opportunity for improving knowledge of the causes of death, knowledge that could ultimately contribute to preventing such deaths. Post-mortem needle biopsies could provide another tool for diagnosing the cause of lethal outbreaks or possibly during surveillance activities for describing pathogen specific burden of mortality. The procedure was deemed much more acceptable than autopsy because it required less time to perform, and therefore did not delay burial, did not produce cuts on the body, and did not remove organs.

According to our respondents, the prerequisites for obtaining consent for this procedure are considerable. First, the research team must be able to quickly acquire the trust of the family and community and based upon that trust, communicate in a compassionate way the potential benefits to society or the family of diagnosing the cause of death. The centrality of building community trust described by our respondents has been reported from other low-income research settings [51,52]. Once trust is established and consent provided, the team must be able to mobilize quickly a trained physician and specimen collection supplies. Major challenges include the short time window in the burial process when the procedure would be acceptable and possible damage to families' trust in health authorities during an outbreak, even prior to arrival of the outbreak investigation team [6].

International guidelines on informed consent in research propose that communicating study methods, risks, and benefits to individuals is of primary importance and that the research team is responsible for communicating these to study participants [53]. However, our respondents concluded that ultimately, the research team alone may be unable to provide complete information about risks and benefits of participation in studies involving post-mortem biopsies. As part of a larger society, guardians and families would require knowledge about relatives' and community and religious leaders' perceptions in order to make a truly informed decision. Similar indigenous, communal informed consent processes have been noted in other low income countries [53]. This kind of shared responsibility offered numerous benefits according to our study participants, including ensuring acceptance of their actions by their society, and some relief from making emotionally taxing decisions in isolation. The indigenous decision-making process and international guidelines for participation in research must both be addressed by researchers [53], although this commitment greatly increases their responsibilities in acquiring informed consent. Researchers must engage not only with guardians, but with families and communities in informed decision-making.

The support for this procedure in these traditional, Muslim communities was somewhat unexpected. One explanation could be that respondents were asked about what they would have done in a particular situation and their responses, however honest, poorly reflect what they would do in reality. A likely contributor of the acceptance of post-mortem needle biopsies in this study was the skill of the highly trained and experienced anthropologists in building rapport and easing participant anxiety during interviews. As respondents frequently mentioned, trust would be an essential consideration in conducting the procedure. The interviewers were likely very capable in building trust with interviewees quickly, due in part to the fact that our research team had prior relationship with these families, which made respondents more accepting of the procedure they proposed. Another explanation for this support could be that respondents valued pleasing the research team, and simply told them what they believed they wanted to hear. While this kind of bias could be real, offers by community and religious leaders to promote the procedure in their communities suggests that their support was genuine. Two outbreak villages that participated in this study were also involved in a follow-up study in 2005 to investigate community residents' understanding of the Nipah virus prevention messages delivered during the outbreak. During those interviews, respondents were critical of researchers involved in the outbreak and the prevention messages that the research team delivered [6]. This suggests that respondent support for post-mortem biopsies in this study may not be entirely due to courtesy bias.

Findings from this study highlight possible harms associated with collecting post-mortem biopsies in Bangladesh. In particular, unequal power relationships could lead some participants to agree to the procedure against their will. According to our respondents, some guardians may not feel empowered to refuse participation in a hospital or if community leaders support it. Some women also reported that they would be unable to refuse participation if their husband agreed. Researchers embarking on this kind of research should be aware of these possible harms and should actively follow-up with participants to better understand and minimize these harms.

## Conclusions

The key to successful collaborations between communities and researchers is trust and good communication, and the issue of post-mortem exam is no exception. The best way to understand the acceptability of post-mortem biopsies in Bangladesh will be to pilot the procedure in the clinical setting with careful follow-up with participants about their experience with decision making and the consent process to minimize potential harms. Findings from this study will be useful in designing protocols to approach and consent families for such a pilot activity. This exploratory study could be duplicated in other settings where additional information about cause of death would be beneficial and where community perceptions about post-mortem exam are unknown. Determining the diagnostic value of post-mortem needle biopsies in determining cause of death in this setting should be a primary goal of future research.

## Competing interests

The authors declare that they have no competing interests.

## Authors' contributions

ESG and SPL conceived of the study; ESG drafted the first protocol; all authors revised the protocol and provided technical input; ESG, SP, and MSI oversaw data collection; SP and MSI collected, summarized, and translated the data; ESG performed the data analysis and drafted the first manuscript; all authors critically revised the manuscript and approved of submitting the manuscript for publication.

## Pre-publication history

The pre-publication history for this paper can be accessed here:

http://www.biomedcentral.com/1472-6939/12/10/prepub
